# Modeling competitive evolution of multiple languages

**DOI:** 10.1371/journal.pone.0232888

**Published:** 2020-05-12

**Authors:** Zejie Zhou, Boleslaw K. Szymanski, Jianxi Gao

**Affiliations:** 1 Department of Computer Science, Rensselaer Polytechnic Institute, Troy, New York, United States of America; 2 Department of Physics, Rensselaer Polytechnic Institute, Troy, New York, United States of America; East China Normal University, CHINA

## Abstract

Increasing evidence demonstrates that in many places language coexistence has become ubiquitous and essential for supporting language and cultural diversity and associated with its financial and economic benefits. The competitive evolution among multiple languages determines the evolution outcome, either coexistence, or decline, or extinction. Here, we extend the Abrams-Strogatz model of language competition to multiple languages and then validate it by analyzing the behavioral transitions of language usage over the recent several decades in Singapore and Hong Kong. In each case, we estimate from data the model parameters that measure each language utility for its speakers and the strength of two biases, the majority preference for their language, and the minority aversion to it. The values of these two biases decide which language is the fastest growing in the competition and what would be the stable state of the system. We also study the system convergence time to stable states and discover the existence of tipping points with multiple attractors. Moreover, the critical slowdown of convergence to the stable fractions of language users appears near and peaks at the tipping points, signaling when the system approaches them. Our analysis furthers our understanding of evolution of various languages and the role of tipping points in behavioral transitions. These insights may help to protect languages from extinction and retain the language and cultural diversity.

## 1 Introduction

Language is for its speaker an essential component of their culture with great importance also for business and economic activities, especially those involving international knowledge transfer [1], interdisciplinary research [2], or international management processes [3]. The dynamic of the language competition has attracted considerable attention in the past decades, resulting in the development of the mathematical models for competition between two languages [4], language acquisition, variation across languages [5], and dynamics of language norm changes [6]. Language competitive dynamics fuel the changes in fractions of speakers of various languages [7], and their collisions [8], blending, and evolution. The final outcome of such evolution can be dominance of one language over the others, the extinction of dominated languages [9], language coexistence, or unification of close languages into one. This process is affected by both internal and external factors. Internal factors represent inherent characteristics of languages, such as lexical and phonological factors [10]. External factors account for social, political and economic influences, such as “The Speak Good English Movement” in Singapore [11] and implementation of standardized Mandarin in China [12]. Both factors influence how people choose their languages and indirectly determine the fraction of speakers of languages, leading to equilibrium with different fractions of speakers for those languages. Languages under the multiple language competitive dynamics may be in one of three different states: (i) dominate state, i.e., entire population only speak this language; (ii) coexistence state, i.e., there are positive fractions of the population using this language; (iii) extinction state, no one speaks this language.

Language dynamics is important for understanding the connections between languages [13] which is related to the language competition and language learning [5], and second language acquisition [14] that supports the existence of bilingual interactive activation [15]. Furthermore, analysis and modeling of language competition can also be extended to social sciences such as population interactions [16], formation of collective opinions [17], cultural evolution [18], increases language and cultural diversity [19], and opinion dynamics [20].

In the past decade, a variety of models have been developed to understand the competitive dynamics of languages. Most of the attention has been paid to the coexistence of two languages [7, 16, 21] and the corresponding bilingualism [22]. One research in this area is finding factors that affect the evolution of competing languages, such as social interaction networks [7], or microscopic competition between two languages [21]. Another research area is modeling abstract competition factors by using real data to estimate the model parameters for transforming language from old to new forms [23] or estimating all parameters in language competition [24]. Moreover, some researches focus on combining the area of language dynamics with the area of statistical physics [20, 25] or applying to them statistical laws to describe word uses [26]. These previous works develop approaches to modeling, analyzing, and even quantifying the competition and evolution of languages, enabling us to theoretically construct and simulate dynamics of two languages. In [27], the authors extend the Abrams-Strogatz model to the competition among multiple languages, but their model becomes very complicated with large number of languages. Still, this multiple language competition model has not been validated on real-word data. Thus, we ask the critical questions: What are the critical parameters that determine the existence and values of tipping points of language coexistence and extinction in the community? And is there a system metric that can indicate the approaching extinction of a language?

Here, we answer the questions raised above by using real-world language evolution data from Singapore [28] and Hong Kong [29–31] to find optimal parameters and validate the extended Abrams-Strogatz model [27]. The model parameters found in this process drive the utilities of competing languages, the strength of majority preference for the most popular language and minority aversion to this language and measure this utility’s impact on language evolution. A combination of factors such as cultural, political, or economical act as an invisible hand [32] in the rise and fall of languages. Our goal is to measure the impact of such factors by representing them as language parameters and establishing their values based on past data on the language evolution. Currently, all the languages modeled here coexist. We investigate the behavioral transitions of the languages by perturbing their parameters. We find that the language with the highest language utility tends to grow faster and eventually gain the largest fraction of speakers. Moreover, when the majority preference is small than a certain critical value, the popular languages may lose its leading position during the evolution process. Finally, when the minority aversion is not sufficiently strong, the languages with small (including the language with the smallest) initial fractions of speakers may gain the largest fraction of speakers. From the above analysis, we obtain the complete phase diagram for each community, showing the evolution of each language in each dataset and the relation between transitions and parameters. Secondly, we analyze the relation between convergence time and state of competing languages, and show that the competition arises to the highest level when the language dominance switches from one language to another. Finally, we illustrate individual and combined effects of two language biases, the majority preference and the minority aversion, by simulating how languages with the largest initial fractions of speakers are affected.

## 2 Methods and data

### 2.1 Data and modeling

To model the real world language competitions, we use dataset of languages used in Singapore (the whole country), languages used in the Chinese community of Singapore, languages used in Indian Community of Singapore, and languages used in Hong Kong. The summary of the data set is given in Table 1. We consider speakers of one language in our dataset as people who consider this language as their primary language.

**Table 1:** We adopt four data sets in our modeling part: Singapore the whole country data set, Chinese Community in Singapore data set, Indian Community data set, and Hong Kong data set from previous work [28, 29, 31] and government statistics [30].

Data Set Name	First Language	Second Language	Third Language	Fourth Language
Singapore	English	Dialect	Mandarin	
Chinese Community	English	Dialect	Mandarin	
Indian Community	English	Tamil	Malay	
Hong Kong	English	Hakka	Hoklo	Sze Yap

#### Singapore

In the 1950s, dialects such as Hokkien were the most widely spoken language in Singapore. In the 1957 census, about 1.8% people mainly spoke English, and about 0.1% people mainly spoke Chinese Mandarin. However, after the implementation of a series of policies from the 1950s to present, the proportions of speakers of different languages in Singapore considerably changed. Until 2010, English and Chinese Mandarin became the most spoken languages with speakers proportion of 32.3%, 35.6%, respectively in Singapore’s entire country [28].

#### Hong Kong

We use language data collected between 1949 to 2016 in Hong Kong [29–31]. The number of people who mainly speak English increased in these 67 years, surpassing the number of people who mainly speak Hakka, Hoklo, or Sze Yap. We do not include Cantonese speakers in our dataset since the number of people who use Cantonese as their common language is much larger than the speaker population in our dataset. We normalize all languages in our dataset before fitting them into our model.

We employ the extended Abrams-Strogatz model [27] to test the competition among multiple languages,

$$\begin{array}{c} \frac{dx_{i}}{dt}=\sum_{j=1,j\neq i}^{n}x_{j}P_{ji}-x_{i}\sum_{j=1,j\neq i}^{n}P_{ij} \end{array}$$ (1)

where *x*_*i*_ is the fraction of the population speaking language *i*, and *P*_*ji*_ *P*_*ij*_ represents the transition rate from language *j* to language *i*.

$$\begin{array}{c} P_{ji}P_{ij}=s_{i}x_{i}^{\beta }{\left(1-x_{j}\right)}^{\alpha -\beta } \end{array}$$ (2)

where *β*(≥0) and *α* − *β*(≥0) represent the strength of the majority preference and the minority aversion, respectively. *s*_*i*_ > 0 is the utility of language *i*, and $\sum_{i=1}^{n}s_{i}=1$.

We utilize numerical simulation to compute the parameters in our model. The following equation calculates the difference:

$$\begin{array}{c} \epsilon D=\sum_{j=1}^{m}\sqrt{\sum_{ti=1}^{n}{\left(x_{ti,j}-x_{ti,j}^{\prime }\right)}^{2}} \end{array}$$ (3)

where *t* is the time step (we use year as a time step unit) varying from 1 to n which is the number of total time steps, *j* is the index of languages varying from 1 to m, which is the number of languages. *x*_*t*,*j*_ is the rational value of fraction of language j users at time step *t*, $x_{ti,j}^{\prime }$ is the theoretical value of a fraction of language j users in time step t.

**Fig 1:**
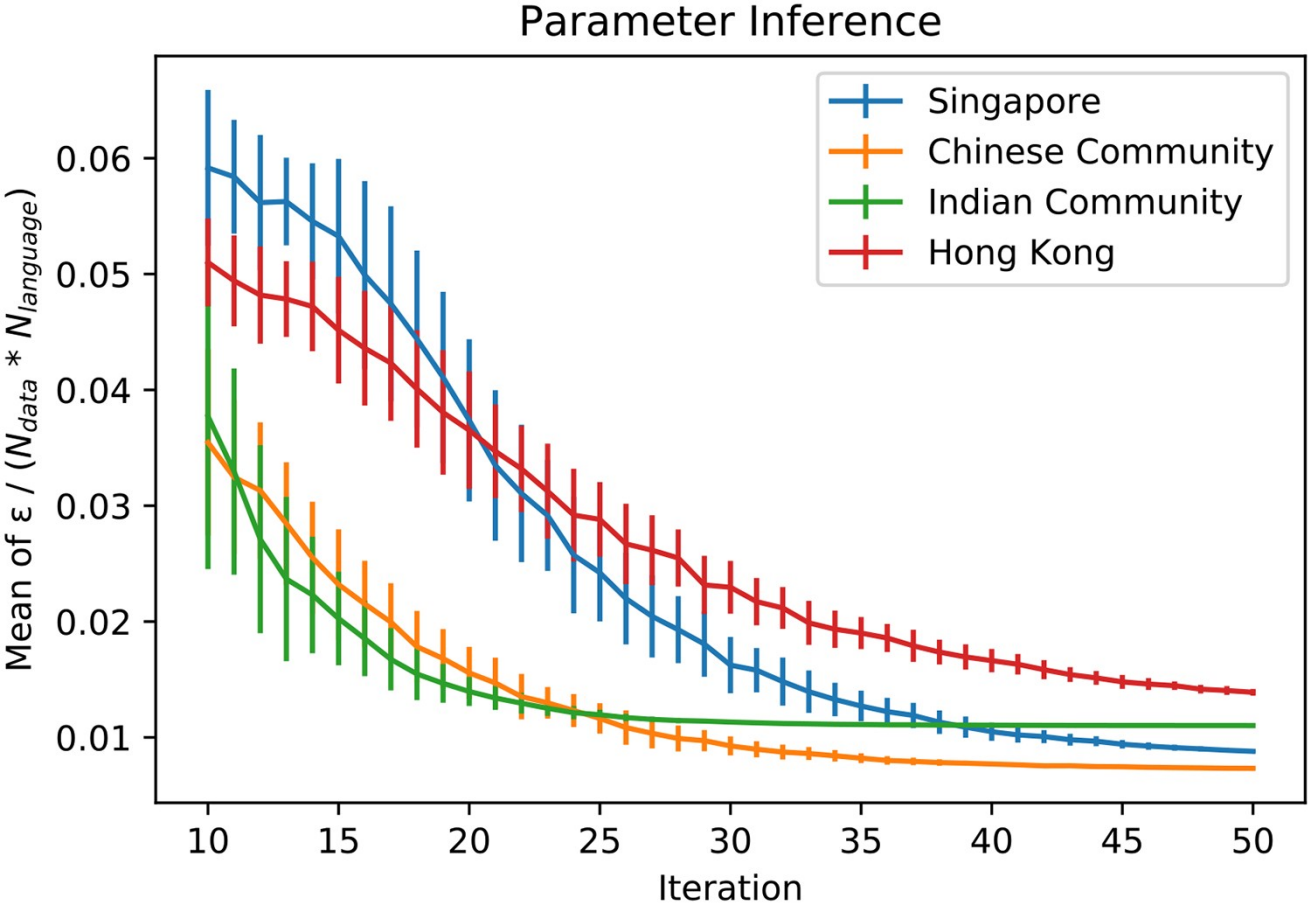
We infer language parameters using the Approximation Bayesian Computation method. Here we plot the values of the epsilon that represents the distance between theoretical and real data for iterations from 10 to 50. The number of particles (aka walkers) is limited to 100. Each particle outputs an *ϵ* representing the distance between theoretical values computed from a set of estimated parameters and target values. *N*_*data*_ represents the number of time points per language in the target data set. *N*_*language*_ represents the number of languages in the target data set. Vertical lines represents the standard deviation of *ϵ*/(*N*_data_*N_*language*_) of the 100 particles in each iteration.

### Model calibration

To fit this model to our language data, we adopt the Approximate Bayesian Computation method implemented as a python package, astroABC and showed the algorithm [33] below:

**Algorithm 1** ABC SMC algorithm for estimating the posterior distribution for parameters *θ* using N particles, the prior distribution *π*(*θ*), given data D and a model for simulating the data M(D|*θ*). *θ*_*i*_,t represents the parameter set for particle i and iteration t.

1: Set the tolerance thresholds, *ϵ*_*t*_ for t = 0 ⋯ T iterations.

2: **procedure** ABC SMC LOOP

3: At iteration *t* = 0:

4:  **for** 1 ≤ *i* ≤ *N* **do**

5:   **while** *ρ*(*D*, D*)>*ϵ*_0_ **do**

6:    Sample θ* from prior θ* ∼ *π*(*θ*)

7:     Simulate mock data D* ∼ *M*(*D*|θ*)

8:     Calculate distance metric *ρ*(*D*, D*)

9:    Set *θ*_*i*,0_ ← θ*

10:    Set weights *w*_*i*,0_ ← 1/*N*

11:   Set covariance $\sum_{0}^{2}\leftarrow 2\sum \left(\theta_{1:N,0}\right)$

12: **for** 0 < *t* ≤ *T* **do**

13:   **for** 1 ≤ *i* ≤ *N* **do**

14:    **while** *ρ*(*D*, D*)>*ϵ*_*t*_ **do**

15:     Sample θ* from previous iteration. θ* ∼ *θ*_1:*N*, *t*−1_ with probabilities *w*_1:*N*, *t*−1_

16:     Perturb θ* by sampling θ** ∼ $N(\theta ,\;\sum_{t-1}^{2})$

17:     Simulate mock data D* ∼ *M*(*D*|θ**)

18:     Calculate distance metric *ρ*(*D*, D*)

19:    Set *θ*_*i*,*t*_ ← θ**

20:    weights $w_{i,t}\leftarrow \frac{\pi (\theta_{i,t})}{\sum_{j=1}^{N}w_{j,t-1}K\left(\theta_{j,t-1}|\theta_{i,t},\sum_{t-1}\right)}$ using kernal *K*

  Set covariance $\sum_{t}^{t}$ using e.g. twice weighted empirical covariance.

We set a distance function for the parameter inference process according to Eq 3. Then, we run the parameter inference until either the maximum number of iterations or the minimum *ϵ* value is reached. In each iteration, 100 particles explore the distances between theoretical values and target values for different sets of parameters. Before terminating, each particle outputs an *ϵ* representing this distance. We plot the results in Fig 1.

**Fig 2:**
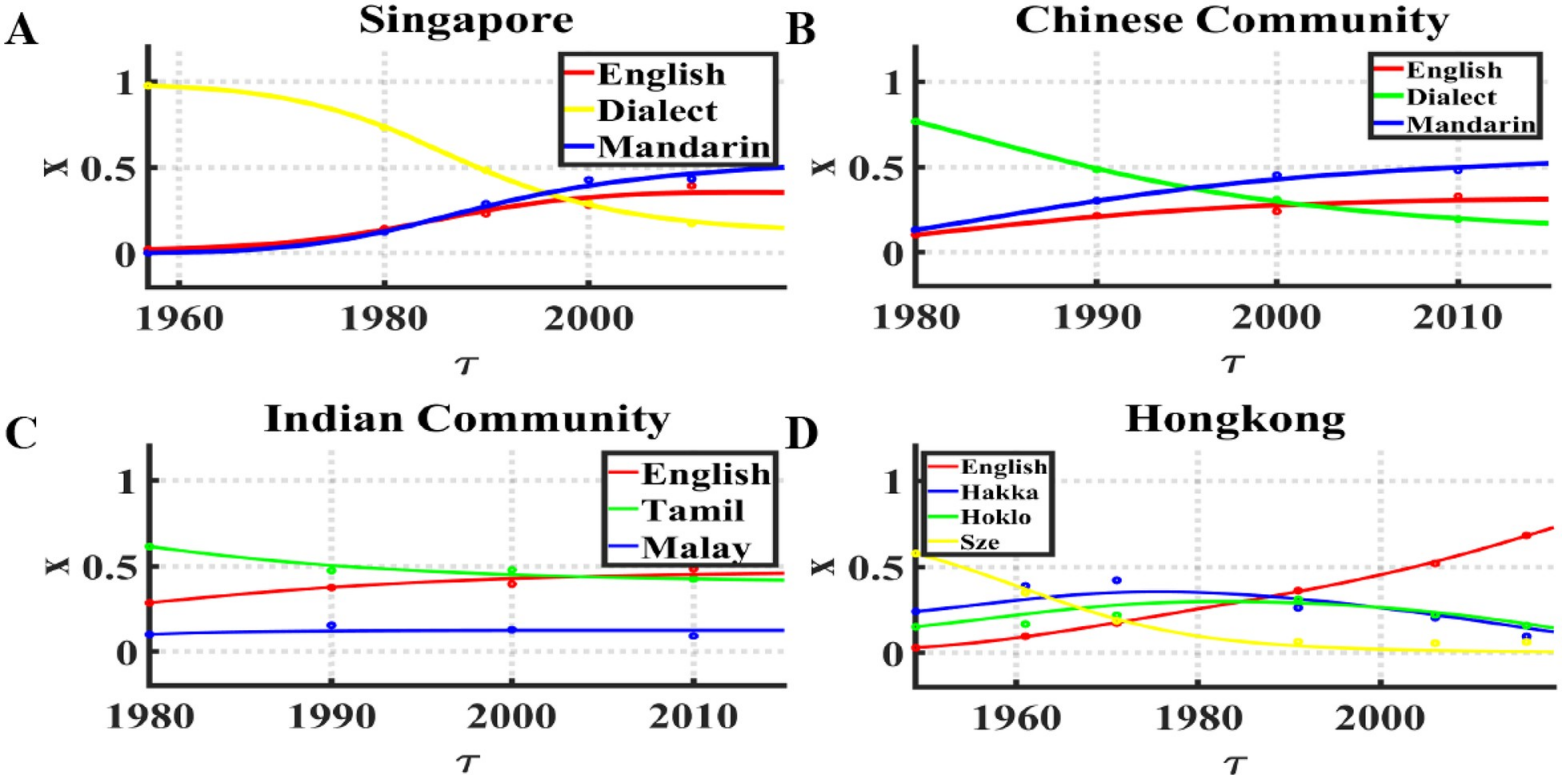
The language evolution over time. Real data are shown by dots, theoretical data are shown by solid lines, real and theoretical data are all normalized. X represents the fraction of language, *τ* represents the time point. (A) Language data of the whole country in Singapore between 1957 and 2010. Parameters fit to dynamic model (*α* ≈ 1.00, *β* ≈ 0.76, English: *s*_*i*_ ≈ 0.35, Dialect: *s*_*j*_ ≈ 0.29, Mandarin: *s*_*k*_ ≈ 0.36), error is *ϵ* ≈ 0.13. (B) Language data of Chinese community in Singapore between 1957 and 2010. Parameters fit to dynamic model (*α* ≈ 0.90, *β* ≈ 0.87, English: *s*_*i*_ ≈ 0.34, Dialect: *s*_*j*_ ≈ 0.29, Mandarin: *s*_*k*_ ≈ 0.37), error is *ϵ* ≈ 0.09. (C) Language data of Indian community in Singapore between 1957 and 2010. Parameters fit to dynamic model (*α* ≈ 1.06, *β* ≈ 0.10, English: *s*_*i*_ ≈ 0.41, Tamil: *s*_*j*_ ≈ 0.40, Malay: *s*_*k*_ ≈ 0.20), error is *ϵ* ≈ 0.13. (D) Language data of Hong Kong between 1949 and 2016. Parameters fit to dynamic model (*α* ≈ 1.21, *β* ≈ 0.90, English: *s*_*i*_ ≈ 0.30, Hakka: *s*_*j*_ ≈ 0.26, Hoklo: *s*_*k*_ ≈ 0.27, Sze Yap: *s*_*l*_ ≈ 0.18), error is *ϵ* ≈ 0.31.

The parameters used by the algorithm are: language utility *s*_*i*_ where the larger the utility, the more attractive and robust that corresponding language is; majority preference *β* which drives the growth of language with large fraction of speakers; minority aversion *α* − *β* which restrains the growth of language with small fraction of speakers. By fitting the language data into extended Abrams-Strogatz model, we compare the real evolution processes in different districts and their corresponding simulation results in Fig 2. It is clear that the model successfully represents the behavior of the data, correctly showing the evolution of each language. In Singapore, the whole country dataset, Dialect starts with about 0.975 fractions of speakers and is surpassed by Mandarin in 1996, by English in 1997. The small minority aversion makes it possible for English and Mandarin, the two languages with small initial fraction, to grow. In addition, the utility of Mandarin is the largest and therefore it attracts the largest fraction of speakers after 100 years. As for Chinese community of Singapore dataset, the trend of speakers in a different language is similar to that of speakers in Singapore the whole country dataset where English and Mandarin gradually replace dialects [34]: it starts with about 0.766 fractions of speakers and is surpassed by Mandarin in 1994, by English in 2001. Here, the minority aversion is also very small so we can observe the growth of English and Mandarin. In the Indian community of Singapore dataset, Tamil started with the most substantial fraction (about 0.613) of speakers but continually lost its speakers and eventually was exceeded by English in 2003. The majority preference is small and the minority aversion is relatively large, leading to the small changes in speakers for all languages. In this case, English increases slowly but it is used by the largest fraction of speakers due to its highest utility. The commonality of these three datasets is the increase of English speakers, which might be caused by the increasing number of English medium schools [28] in this period. The expanding usage of Mandarin in Singapore the whole country dataset and Chinese community dataset might benefit from “Speak Mandarin Campaign” implemented in 1979 in Singapore [35]. In Hong Kong dataset, Sze Yap, which is a Chinese vernacular in Hong Kong, owns the most substantial fraction of speakers(about 0.578) at the beginning but is gradually replaced by the remaining three languages, and it almost went to extinction in 1999. The minority aversion here is also very small, allowing English, the language with the largest utility, to grow really fast.

### 2.3 State definition

We will refer to the language with the largest number of speakers as the most popular, but if this language drives the competing languages to extinction, we will refer to is as dominant.

**Fig 3:**
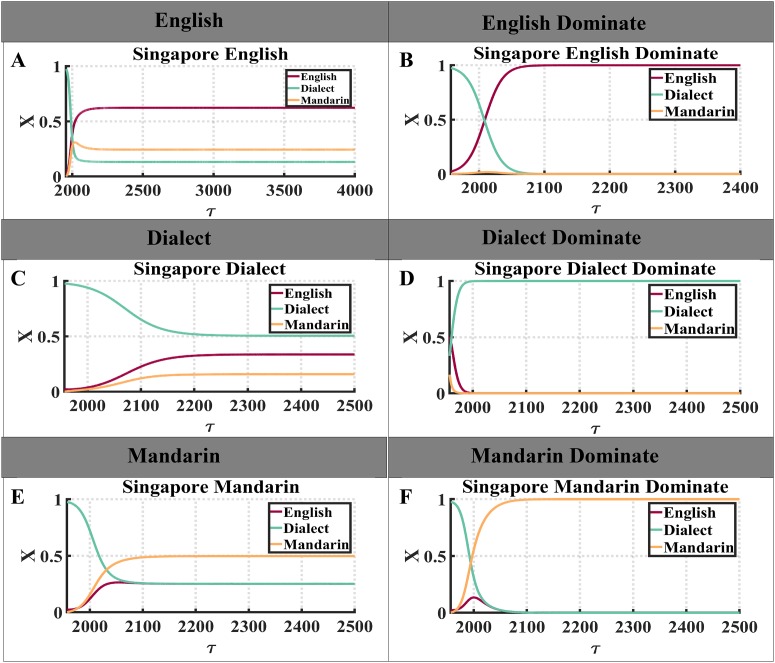
We use Singapore dataset to illustrate “coexistence state” and “dominance state” of a language. “X” represents the fractions of languages and *τ* represents time steps. (A) After *τ* = 2500, competing languages stay in coexistence state with English acquiring the largest fraction of speakers. (B) English is dominant since from *τ* = 2100, the fractions of all other languages decrease to an extremely low level. (C) Dialect gets the largest fraction of speakers without leading to the extinction of Dialect or Mandarin. (D) Dialect’s fraction of speakers increase to an extremely high level, leading to the extinction of other languages. (E) Mandarin’s fraction of speakers grows and surpass English and Dialect. Eventually, all languages stay in coexistence state and Mandarin owns the largest fraction of speakers. (F) As Mandarin increases its number of speakers significantly, other competing languages go extinct.

Here we need to define each state in the evolution of language competition. In our simulations, two distinct states are defined as: “coexistence state” and “dominance state”. “Coexistence state” arises if at least two languages survive but in this state, an extinction of some languages is still possible. In contrast, in “dominance state”, the survival of one language leads to the extinction of all others. The “coexistence state” and “dominance state” are illustrated in Fig 3, where the left column shows example of “coexistence state” with its title showing the language with largest fraction of speakers, while the right column displays examples of “dominance state”. Note that the “coexistence state” and “dominance state” here all refer to the state after fractions of speakers for all languages converge to their steady states.

As shown in Fig 3A, during language competition, Dialect’s fraction of speakers drops dramatically, while English’s fraction of speakers increases. Eventually, none of the competing languages disappear, with English being the most popular language. In contrast, English is shown to be the only survivor after the language competition in Fig 3B, because English gains a substantial fraction of speakers while other languages lose almost all theirs. In Fig 3C, Dialect loses a large number of speakers in the competition, causing its competitors to gain more speakers, but it still holds the most speakers and coexists with other competing languages. Dialect surpasses English and becomes dominant in Fig 3D, where fractions of speakers of languages other than Dialect all reduce to a shallow level. Similarly in Fig 3E, Mandarin owns the largest fraction of speakers and stays in “coexistence state,” while in Fig 3F, Mandarin leads to the extinction of all other languages. As we took the Singapore dataset as an example to define different states, we are now able to describe future simulations in a more straight forward way.

## 3 Results

### 3.1 State utility *s*_*i*_

In the extended Abrams-Strogatz model, *s*_*i*_ represents the utility of language *i*. Accordingly, in our language dataset, each language *i* has its own *s*_*i*_ representing its utility. We analyze the relation between *s*_*i*_ and the competition between language *i* and other languages when *s* is in the range [0, 0.6]. Since the total of all language utilities is by definition 1, in each simulation, with the increasing of the utility of one language, increasing utility of one language decreases utilities of other languages proportionally to their current utility.

**Fig 4:**
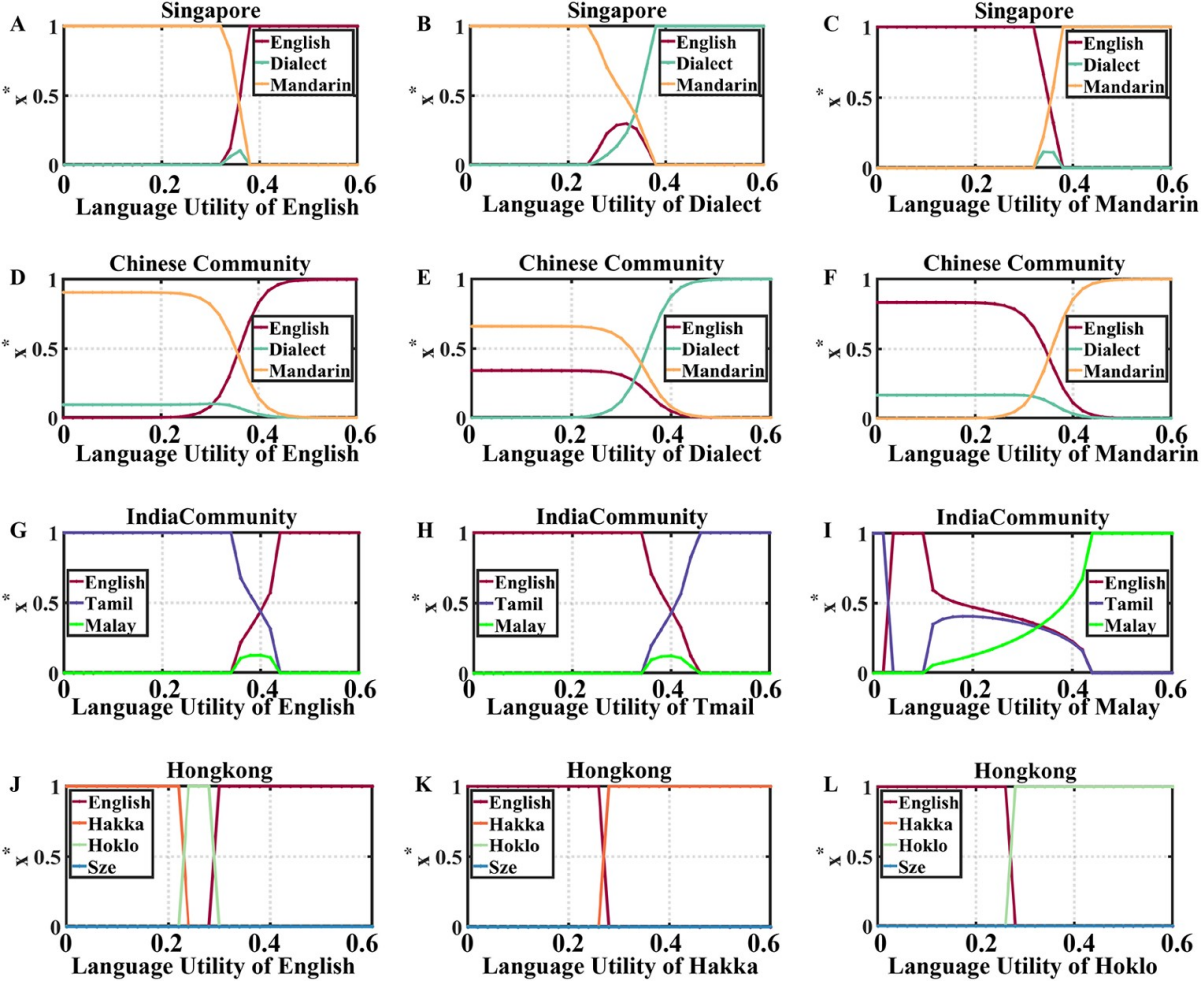
Fraction of language speakers in relation to this language utility, where X* represents the fraction of a language users at the steady state of competition, and *s* stands for language utility. (A) Fraction of language speakers in relation to the English utility in the Singapore whole country dataset. (B) Fraction of language speakers in relation to Dialect utility in the same dataset. (C) Fraction of language speakers in relation to Mandarin utility in the same dataset. (D) Fraction of language speakers in relation to English utility in Chinese community in Singapore. (E) Fraction of language speakers in relation to Dialect utility in Chinese community in Singapore. (F) Fraction of language speakers in relation to Mandarin utility in Chinese community in Singapore. (G) Fraction of language speakers in relation to English utility in Indian community in Singapore. (H) Fraction of language speakers in relation to Tamil utility in Indian community. (I) Fraction of language speakers in relation to Malay utility in Indian community. (J) Fraction of language speakers in relation to English utility in Hong Kong. (K) Fraction of language speakers in relation to Hakka utility in Hong Kong. (L) Fraction of language speakers in relation to Hoklo utility in Hong Kong.

In the Singapore whole country dataset (Fig 4A, 4B and 4C), we set majority preference *β* ≈ 0.76 and minority aversion *α* − *β* ≈ 0.24. For Fig 4A, when *s*_*i*_ ∈ [0, 0.32], languages are in “dominance state” with Mandarin being dominant. As *s*_*i*_ further increases, languages come into “coexistence state” for a short while, and the fraction of English speakers surpasses the fraction of Mandarin speakers for *s*_*i*_ ≥ 0.36. When *s*_*i*_ > 0.38, English becomes dominant and drives others to extinction. In Fig 4B, language competition starts in “dominance state” as well (language utility of Dialect is in the range *s*_*j*_ ∈ [0, 0.24] and Mandarin is dominant. Then, the system reaches “coexistence state” in which all competing languages’ fractions change dramatically. When the utility of Dialect exceeds 0.38, it becomes dominant. Similarly, in Fig 4C, in the beginning, when Mandarin utility is in the range *s*_*k*_ ∈ [0, 0.32], English is dominant. Then, for *s*_*k*_ ∈ [0.34, 0.36], the system is in “coexistence state” and the fraction of English speakers drops significantly. Eventually, Mandarin becomes dominant when its utility exceeds 0.38.

In Chinese community dataset (Fig 4D, 4E and 4F), we set majority preference *β* ≈ 0.87 and minority aversion *α* − *β* ≈ 0.03. In Fig 4D, Three languages are in “coexistence state”. When language utility of English is in the range *s*_*i*_ ∈ [0, 0.45], then English starts to dominate the others. In Fig 4E, when language utility of Dialect is in the range *s*_*j*_ ∈ [0, 0.48], three languages coexist, but then Dialect becomes dominant. In Fig 4F, three languages coexist when language utility of Mandarin is in the range *s*_*k*_ ∈ [0, 0.48]. When *s*_*k*_ ≥ 0.5, Mandarin dominates the other languages.

In Indian community dataset (Fig 4G, 4H and 4I), we set majority preference *β* ≈ 0.10 and minority aversion *α* − *β* ≈ 0.97. In Fig 4G, when language utility of English *s*_*i*_ ∈ [0, 0.34], Tamil, which is the language with the largest utility, is dominant. Then the system reaches “coexistence state” as the utility of English further increases. When *s*_*i*_ exceeds 0.44, English starts to be dominant. In Fig 4H, when language utility of Tamil is in the range *s*_*j*_ ∈ [0, 0.34], English is dominant. When *s*_*j*_ ∈ [0.36, 0.44], the system is in “coexistence state”. When *s*_*j*_ > 0.44, Tamil becomes dominant. In Fig 4I, Tamil is dominant when language utility of Malay is in the range *s*_*k*_ ∈ [0, 0.02]. Then, dominance switches to English when *s*_*k*_ ∈ [0.04, 0.1]. When *s*_*k*_ ∈ [0.12, 0.42], all languages come to “coexistence state”. When *s*_*k*_ ≥ 0.44, Malay is dominant.

In Hong Kong dataset (Fig 4J, 4K and 4L), we set majority preference *β* ≈ 0.90 and minority aversion *α* − *β* ≈ 0.32. In Fig 4J, when language attraction of English is in the range *s*_*i*_ ∈ [0, 0.22], Hakka is dominant. The dominant language switches to Hoklo when *s*_*i*_ ∈ [0.24, 0.28]. Then Hoklo and English’s fractions of speakers change dramatically, leading to the dominance of English. In Fig 4K, when language attraction of Hakka is in the range *s*_*j*_ ∈ [0, 0.26], English is dominant. Then, as language utility of Hakka exceeds 0.26, Hakka becomes dominant. Similarly, in Fig 4L, in the beginning, English is dominant, but for *s*_*k*_ ≥ 0.28, Hoklo becomes dominant.

From these simulations, we find that when the language utility *s*_*i*_ of language *i* is relatively small, one of the other languages, which is usually the language with the highest language utility, tends to be dominant. As *s*_*i*_ increases, languages might come into “coexistence state,” which acts as a transition period for language *i* to become dominant. Accordingly, when *s*_*i*_ is large enough, language *i* becomes dominant.

### 3.2 Convergence time

**Fig 5:**
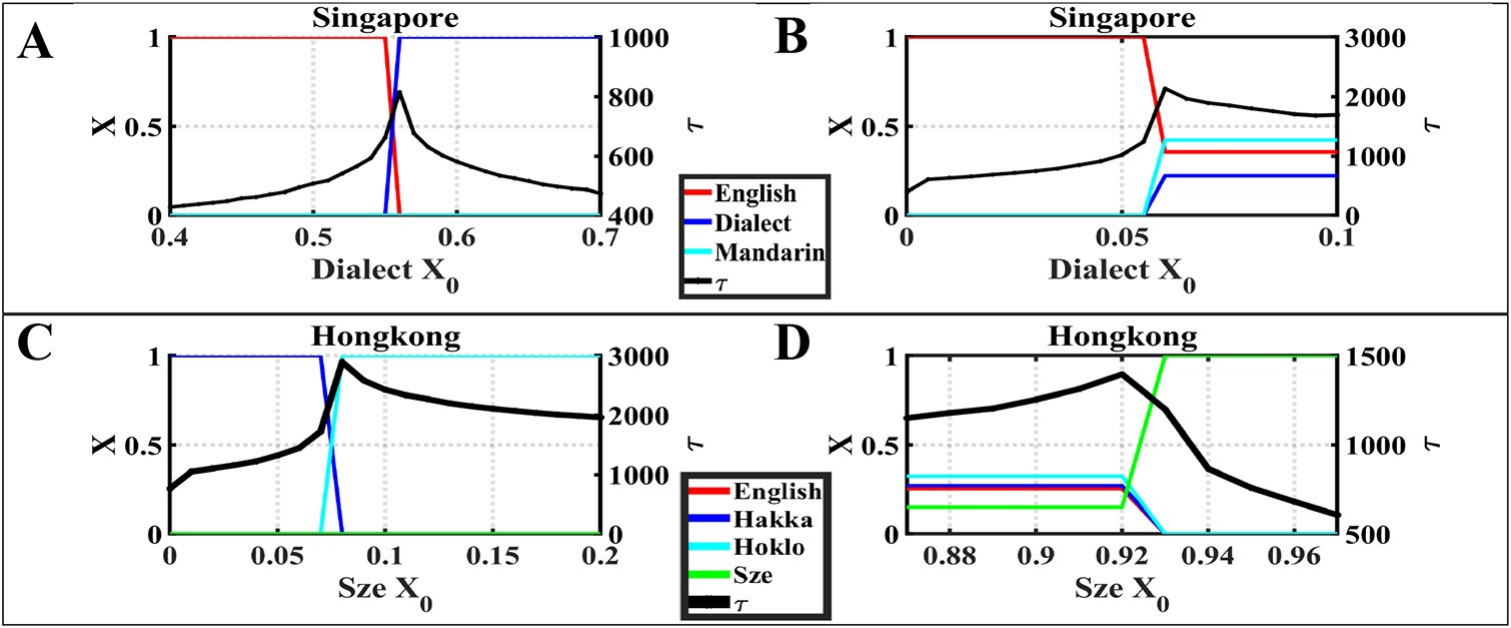
We gradually increase the initial fraction of one language to observe the relation between times used for all languages to reach the steady state of their fractions of speakers and the distance to the tipping point for the initial fraction. (A) When the language dominance switches from one language to another, time to achieve the steady state of each language speakers’ fractions reaches a peak. (B) Similar to subfigure (A), in Hong Kong dataset, when the language dominance switches from one language to another, time to achieve the mentioned above steady state reaches a peak. (C) When the system transitions from ‘coexistence state’ to ‘dominance state’, time to reach the mentioned above steady state again reaches a peak. (D) Before the system transitions from ‘dominance state’ to ‘coexistence state’, time to achieve the mentioned above steady state reaches a peak.

It is notably hard to predict the critical transition from one state to another because the state of the system may show little change before the tipping point [36, 37]. Critical slowdown [38] defined in statistical physics is an indicator for early warning signals with applications to many fields, ranging from the economy [39] to ecology [40]. Here we employ the convergence time as the early warning signals for the behavioral transition in the language competition. Fig 5 shows the convergence time of different datasets under different language competitions, where the *x*-axis represents the initial fraction of one language, the left *y*-axis represents the equilibrium fraction of each language, and the right *y*-axis represents the convergence time *τ*. In Fig 5A, *τ* reaches a peak when the initial fraction of Dialect increases to 0.56 and Dialect replaces English as dominant language. Similarly, at *τ* in Fig 5B, when the system transitions from “dominance state” to “coexistence state”, *τ* reaches its peak. In Hong Kong datasets, we find similar outcome. The convergence pattern observed in Fig 5C is similar to the one seen in Fig 5A since the peak of *τ* happens when the dominance switches from one language to another. As for Fig 5D, the peak of *τ* happens when the system transitions from “coexistence state” to “dominance state,” which is exactly opposite to the transition in Fig 5B, yet they show similar patterns of convergence time.

From these simulations, the peak of convergence time happens when the state transition happens of either switching the dominant language or moving from “coexistence state” to “dominance state,” or vice versa. Such transitions can be caused by the comparable competing strength of different languages. The convergence time enables us to identify the “tipping point” of the system parameter values giving the control system enough time to prevent unwanted transition.

### 3.3 Sensitivity to majority preference and minority aversion

Here, we focus on how majority preference and minority aversion can affect language competitions. With one exception, for each dataset, we set all parameters and languages’ initial fractions of speakers to the values used in modeling and data section. The exception are the values of majority preference *β* and of minority aversion *α* − *β*.

**Fig 6:**
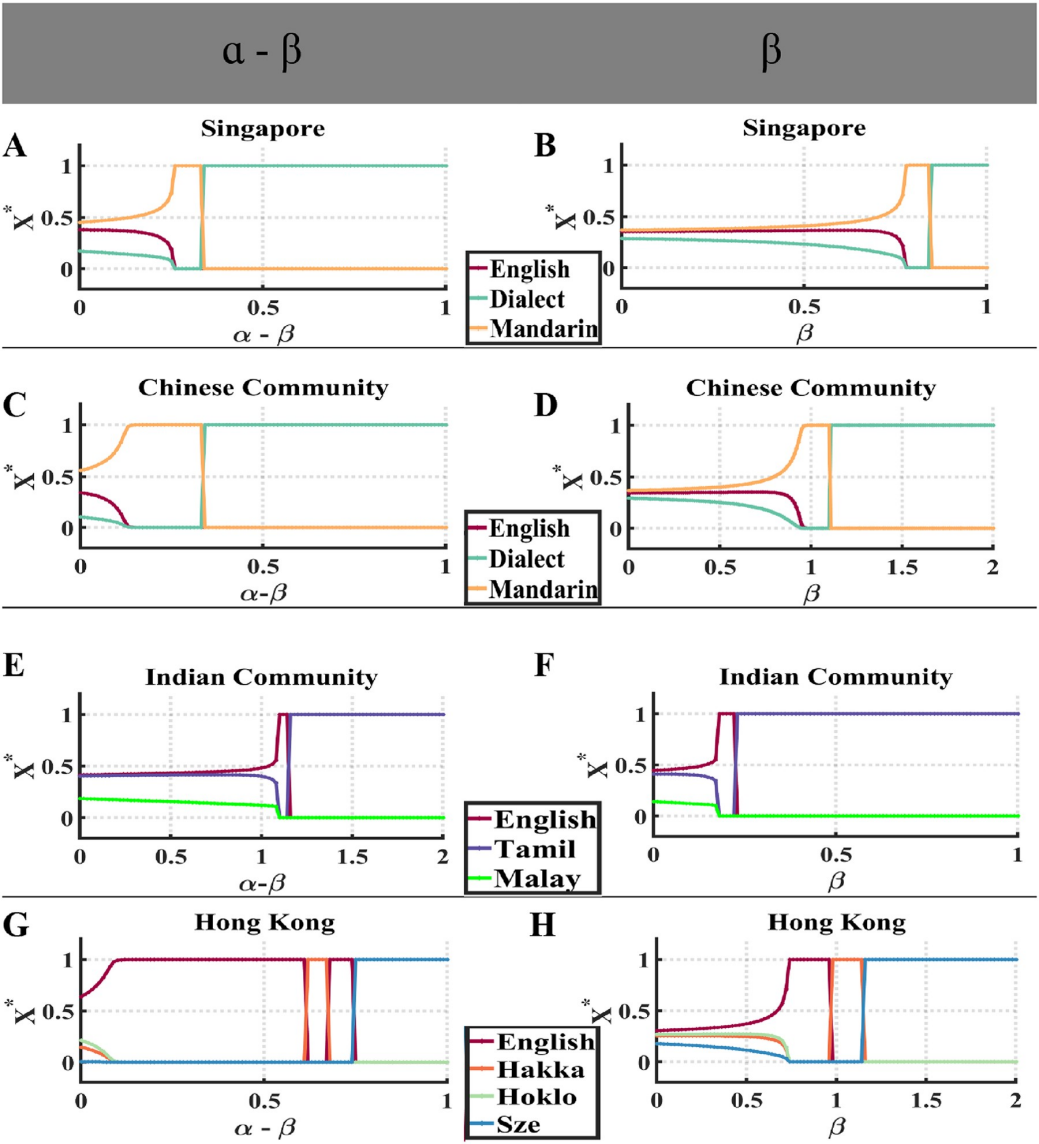
The change of equilibrium point when *α* − *β* or *β* change in each language dataset. (A) The relation between *α* − *β* and fraction of each language in the Singapore whole country dataset. (B) The relation between *β* and fraction of each language in the Singapore whole country data set. (C) The relation between *α* − *β* and fraction of each language in Chinese community in Singapore. (D) The relation between *β* and fraction of each language in Chinese community in Singapore. (E) The relation between *α* − *β* and fraction of each language in Indian community in Singapore. (F) The relation between *β* and fraction of each language in Indian community in Singapore. (G) The relation between *α* − *β* and fraction of each language in Hong Kong. The relation between *β* and the fractions of each language speakers in Hong Kong.

In Fig 6A, we have *β* ≈ 0.76 while *α* − *β* varies from 0 to 1 with step 0.01. In the range of *α* − *β* ∈ [0, 0.25], Mandarin has the largest fraction of speakers, and all languages are in “coexistence state”. In the range of *α* − *β* ∈ [0.26, 0.33], Mandarin is dominant, causing the extinction of Dialect and English. When the minority aversion starts to exceed 0.34, then Dialect, which is the language with the largest initial fraction in this data set, starts to be dominant. In Fig 6C, in the range of *α* − *β* ∈ [0, 0.13], the system stays in “coexistence state” and English is the most spoken language. When *α* − *β* further increases, English is still the most spoken language and dominant. However, as the minority aversion becomes large enough, language with the largest initial fraction (Dialect) of speakers starts to be dominant. In Fig 6E, the system starts in “coexistence state” and English, which is the language with the largest language utility (*s* ≈ 0.41), is the most spoken language in the range of *α* − *β* ∈ [0, 1.08]. Then, when *α* − *β* ∈ [1.1, 1.14], English is again dominant. Tamil, the language with the largest initial fraction of speakers, is dominant when the minority aversion is larger than 1.14. In Fig 6G, when *α* − *β* ∈ [0, 0.1], English, which is the language with the largest language utility (*s* ≈ 0.30), is most popular, but the system is in “coexistence state”. When *α* − *β* ∈ [0.11, 0.61], English is again dominant. When *α* − *β* ∈ [0.62, 0.74], Hakka and English compete with each other for dominance, with Hakka is dominant when *α*−*β* ∈ [0.62, 0.67] and English is dominant when *α* − *β* ∈ [0.68, 0.74]. This may be caused by the two languages’ similar level of competitiveness determined by both initial fraction and utility. However, when minority aversion is high enough (larger than 0.74), Sze Yap, which is the language with the highest initial fraction, becomes dominant.

From these simulations, we conclude that low value of minority aversion favors the growth of languages with small initial fraction, and usually the language with the largest language utility *s* among them, can gain the largest fraction of speakers. In contrast, high minority aversion favors the growth of languages with the largest initial fraction of speakers.

In Fig 6B we set the minority aversion *α* − *β* ≈ 0.24, and vary the majority preference *β* from [0, 1]. Similar to language patterns in Fig 6A, languages start in “coexistence state”, with Mandarin owning the largest fraction of speakers when *β* ∈ [0, 0.77]. When the majority preference increases to (*β* ∈ [0.78, 0.84]), Mandarin becomes dominant, even though it had the smallest initial fraction of speakers. As the majority preference further increases and until it reaches 0.85, Dialect, the language with the largest initial fraction, starts to be dominant.

In Fig 6D, when *α* − *β* ≈ 0.03 and majority preference *β* varies from [0, 2]. The system stays in “coexistence state”, with Mandarin being dominant in the range of *β* ∈ [0, 0.95]. Then as *β* increases to (*β* ∈ [0.96, 1.1]), Mandarin is dominant for a short range of *β* because for *β* > 1.1, Dialect, the language with the smallest initial fraction, is dominant. In Fig 6F, at the beginning with (*β* ∈ [0, 0.17]), English, which is the language with the largest language utility, has the largest fraction of speakers and all languages are in “coexistence state.” For *β* ∈ [0.18, 0.22], English is dominant, but for larger *β*, Tamil becomes dominant. Similarly, as shown in Fig 6H, the system transitions from “coexistence state” to “dominance state”, and the language with the largest initial fraction becomes dominant when majority preference is high enough; in this case, for *β* ∈ [0.74, 0.96] English is dominant, while for *β* ∈ [0.98, 1.14], Hakka takes over this role. Finally, when *β* > 1.14, Sze Yap, the language with the largest initial fraction, is dominant.

From these four simulations, we find that when majority preference is small, languages with small initial fraction (usually the language with the largest language utility) might own the largest fraction of speakers, and becomes dominant. However, when majority preference is high enough, the language with the largest fraction of speakers usually becomes dominant, driving other language to extinction.

**Fig 7:**
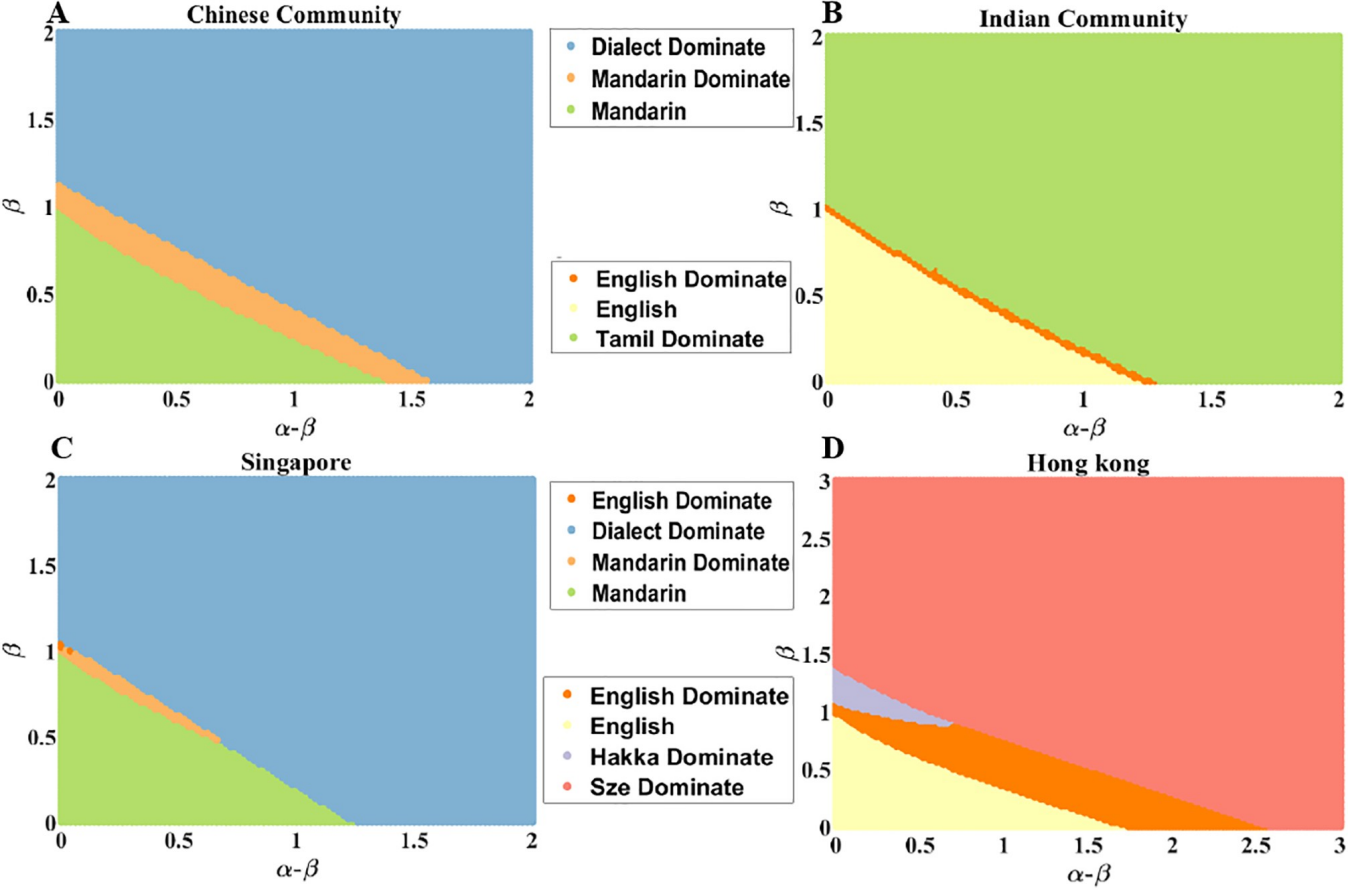
States of the language with the largest fraction of speakers as a function of increasing the majority preference and the minority aversion. “Dominate” denotes one language domination over others, otherwise, all languages shown in the figure coexist. (A) The language with the largest fraction of speakers in relation to *α* − *β* and *β* in Chinese community in Singapore. (B) The language with the largest fraction of speakers in relation to *α* − *β* and *β* in Indian Community in Singapore. (C) The language with the largest fraction of speakers in relation to *α* − *β* and *β* in the Singapore whole country data set. (D) The language with the largest fraction speakers in relation to *α* − *β* and *β* in Hong Kong.

### 3.4 Phase diagram

As for the combined effect of majority preference and minority aversion, we still use the initial language fraction of speakers and parameters from the data and modeling section. Here, we only consider the language with the largest fraction of speakers. In Fig 7A, when majority preference *β* ∈ [0, 0.94], three different kinds of patterns appear. In the first pattern, all three languages, English, Dialect, and Mandarin, coexist and Mandarin is the most popular. The next pattern has Mandarin dominant. The third pattern has Dialect dominant. In this case, when the minority aversion *α* − *β* is large enough, Dialect, which is the language with the most speakers, is dominant. As *β* increases from 0 to 0.94, Dialect becomes more and more likely to become dominant because the range of *α* − *β* for Dialect to be in this role becomes larger and larger. When *β* ∈ [0.96, 1.08], two different patterns arise. In the first, Mandarin is dominant while in the second, it is Dialect which is dominant. When *β* exceeds 1.08, Dialect is dominant. Fig 7B shows that for the majority preference *β* ∈ [0, 0.94], again three different patterns arise. In the first one, three languages, English, Tamil, and Malay, coexist, and Tamil is the most popular. In the second pattern, English is dominant, while in the third it is Tamil that is dominant. Similar to Fig 7(A), the language with the largest utility (Mandarin in Fig 7A and English in Fig 7B) tends to be the most popular when the majority preference and minority aversion are small. With *β* ∈ [0.96, 0.98], two different patterns are present. In the first, again three languages coexist, and English is the most popular. The second has English dominant. The third pattern arises when *β* > 0.98, Tamil, which is the language with the largest initial fraction of speakers, is dominant.

Fig 7C shows that when minority aversion *α* − *β* ∈ [0, 0.04], four different patterns arise. In the first, again three languages, English, Dialect, and Mandarin, coexist and Mandarin has the most speakers. The second pattern has Mandarin as dominant, while in the third pattern Dialect is dominant. Finally in the fourth case it is English that is dominant. In this case, even English can be dominant over a short range of parameters, because of its comparably high initial fraction and language utility; it has the second largest initial fraction and the second largest language utility. With *α* − *β* ∈ [0.06, 0.66], the first three patterns from the case of lowest *β* reappear. Again, Mandarin is the language with the largest utility and it is the most when minority aversion and majority preference are small. When *α* − *β* is greater than 1.24, Dialect is dominant. In Fig 7D, with minority aversion *α* − *β* ∈ [0, 0.68], four different patterns arise. In the first, four languages, English, Hakka, Hoklo, and Sze Yap, coexist, and English is the most popular. In the second, English is dominant while in the third it is Hakka that is dominant. Finally, in the fourth, Sze Yap is dominant. When *α* − *β* ∈ [0.7, 1.72], the previous patterns, the first, the second, and the fourth reappear. When *α* − *β* > 2.56, Sze Yap dominates all other languages. In this data set, English has the largest language utility and owns the largest fraction of speakers when the majority preference and minority aversion are small.

When the majority preference and the minority aversion are relatively small, competing languages tend to coexist with each other and language with the largest utility tends to own the most speakers. Hence, when the majority preference and the minority aversion are relatively small, they affect language competition weakly. It is the language utility that plays an essential role in this competition. As the majority preference and the minority aversion increase, some languages become dominant. When the majority preference and the minority aversion further increase, the language with the largest initial fraction is dominant, indicating that when the majority preference and the minority aversion are large enough, they will favor the growth of the language with the most substantial initial fraction of speakers and make it dominant.

## 4 Discussion

Here we provide a model and its validation using four real-world language competitions involving several languages that extend the Abrams-Strogatz model in the critical direction. The model fits well with the real data, as shown in the first section, enabling us to analyze further factors affecting the language competition in detail. Our contributions can be summarized as follows.

1. We show that language utility affects the competitive evolution of communities using several languages. When the utility of a language is low enough, it might go extinct, but when this utility is high enough, it can be dominant and drive other languages to extinction. However, it is also possible that with the value of this utility in mid-range, the system can be in a “coexistence state”.
2. The relation between convergence time and state transition of languages shows that convergence time to steady state fractions of language users reaches a peak at the state transition tipping points. Such critical slowdown can be caused by similar competitiveness of the different competing languages. At the tipping points either one dominant language is replaced by another or the system transitions from a “dominance state” to “coexistence state” or vice versa.
3. We demonstrate the influence that the majority preference and the minority aversion can separately exert on competing languages. When majority preference is small, a language with small initial fraction of speakers (including the language with the tiniest initial fraction of speakers) can have the most speakers after the language competition, and even lead to the extinction of other languages. When the majority preference is large enough, the language with the largest initial fraction of speakers will win the language competition, usually driving all other languages to extinction. The simulations with varying the minority aversion yield similar results as described above.
4. We also discuss the influence that the majority preference and the minority aversion can together exert on the evolution of competing languages. When both of these preferences are relatively low, a language with the small initial fraction of speakers can gain the most speakers in the steady state, and become dominant. When both biases are high enough, the language with the most speakers initially is most likely to be dominant. Moreover, there are variants of these conditions yielding results other than the two reported above, as shown in Fig 7C where English is dominant as shown in Fig 7D where Hakka is dominant.

Our simulations illustrate the results of the language competition under various conditions, providing examples of the impact of the language utility, the majority preference, and the minority aversion on the competition outcomes. Yet, analytical formulas defining the competitive evolution of languages quantitatively as a function of time and different parameters of the model are not known. Moreover, our simulations are completed without considering geographical [41], physiological [42], and other factors. Hence, constructing a final formulation of language competition in the real world requires future research.

## Supporting information

S1 Table Language data sets in different areas. (XLSX)
